# Effect of night shift work on metabolic syndrome in adults who suffered from earthquake stress in early life

**DOI:** 10.3389/fpubh.2023.1139113

**Published:** 2023-07-20

**Authors:** Na Li, Ran Wang, Peihua Hu, Wenting Lu, Xiaochuan Zhao, Lan Wang, Mei Song, Yuanyuan Gao, Cuixia An, Shahid Bashir, Xueyi Wang

**Affiliations:** ^1^Department of Psychiatry, The First Hospital of Hebei Medical University, Shijiazhuang, China; ^2^Mental Health Center, Hebei Medical University, Shijiazhuang, China; ^3^Clinical Medical Research Center for Psychiatric and Psychological Disorders of Hebei Province, Shijiazhuang, China; ^4^Saudi Arabia Neuroscience Center, King Fahad Specialist Hospital, Dammam, Saudi Arabia

**Keywords:** pregnancy, infancy, earthquake stress, metabolic syndrome, night shift work, adulthood

## Abstract

**Objective:**

To examine the role of night shift work on the risk of metabolic syndrome (MetS) in adults suffered from earthquakes prenatally or as infants and to analyse the effect of stress on factors that influence MetS in this population.

**Methods:**

We included 870 subjects from 2014 to 2015. All subjects work as miners for the Kailuan Mining Group and were born were living in Tangshan. Participants were classified into two groups on basis of their work schedules: day shift and night shift. They were further classified into the prenatal exposure group, the infancy exposure group, and the control group based on their age during the Tangshan earthquake. This study was conducted 38 years after the earthquake. Participants’ general demographic data, smoking and drinking habits, as well as work schedules were collected. All participants’ sleep status was assessed with the Pittsburgh Sleep Quality Index. The measurement of all subjects’ waist circumference and blood pressure was made, and triglycerides, fasting blood glucose, high-density lipoproteins, and low-density lipoproteins were measured by collecting blood samples. The definition of MetS was made after the guidelines for preventing and controlling type 2 diabetes in China (2017 Edition).

**Results:**

A total of 187 (21.5%) workers were determined to have MetS. The incidence of MetS was greatly higher in night shift workers who were exposed to an earthquake during infancy than in day shift workers (*χ^2^* = 8.053, *p* = 0.005). A multivariate logistic regression analysis displayed male participants had a higher risk develop MetS than female participants (*p* = 0.042, *OR* = 0.368, *95% CI* = 0.140, 0.965). Current smokers (*p* = 0.030, *OR* = 1.520, *95%CI* = 1.042, 2.218) and participants who sleep fewer than 7 h per night (*p* = 0.015, *OR* = 1.638, *95%CI* = 1.101, 2.437) had a higher risk of MetS. Prenatal earthquake stress was also a risk element for MetS (*p* = 0.012, *OR* = 1.644, *95%CI* = 1.115, 2.423).

**Conclusion:**

The risk of MetS is significantly higher in night shift workers exposed to earthquake stress during infancy than day shift workers. Earthquake exposure during pregnancy is an independent risk factor for MetS. Smoking and sleeping less than 7 h have a higher risk of MetS than the control group.

## 1. Introduction

Metabolic syndrome (MetS) is defined as a pathologic condition with the characteristics such as abdominal obesity, insulin resistance, hypertension, and hyperlipidaemia by the WHO. These factors are correlated with a higher risk of type 2 diabetes, cardiovascular disease, and mortality. MetS is estimated to affect about 25% of the global population (1). More importantly, MetS -related disorders account for two-thirds of deaths caused globally by non-communicable diseases (NCDs) (2). In these years, it has been estimated that the prevalence of MetS among adults in the US is at around 34.7% (3). The prevalence of MetS among younger individuals (aged 20–39 years) has recently increased significantly (from 16.2 to 21.3%)—especially among older individuals between 2011 and 2016 (3). More than 4/5 of adults had at least one abnormal MetS component in China (4). Individuals who are younger when they first develop MetS show a higher risk of also developing cardiovascular disease (5).

Both genetic and environmental elements such as the in the utero environment, exert significant effects on the growth of MetS. Several studies have found that the risk of MetS increases following stress in early life (6, 7). The Dutch Famine Birth Cohort Study found that the children born to women who suffered famine during pregnancy are more likely to have several symptoms of MetS including insulin resistance, dyslipidaemia, obesity, and hypertension (8). Men exposed to two or more psychosocial traumatic events and women exposed to one psychosocial traumatic event have a higher risk of MetS than individuals who are not exposed to trauma (9). Doom and colleagues (10) found that a worse psychosocial environment during infancy is associated with higher blood pressure, a higher risk of biomarkers related to MetS (greater triglycerides and cholesterol), and a higher risk of MetS in adolescence.

The assumption that factors during early life (preadult), such as exposure to famine, may exert an effect on the pathophysiology of metabolic diseases in adulthood has attracted basic study interest especially in the last two decades (11, 12). Our previous research on earthquake stress found that earthquake stress is an independent risk factor with long-term impacts on diabetes incidence (13). Our previous research also showed that earthquake exposure during infancy is a great independent risk element for hypertension (14). Previous studies on earthquakes and health have been short-term follow-up studies, and no studies have investigated early life earthquake exposure to MetS in adulthood (15, 16). This study base on our previous research to investigated the effect of earthquake stress in MetS in adulthood after 38 years of maternal and fetal exposure to earthquake stress.

Lifestyle factors such as sleep disturbance, daily calorie intake, alcohol consumption, and smoking are related to the risk of MetS (4, 17). Humans spend approximately one-third of our lives sleeping. Sleep plays a crucial role in the body’s energy metabolism. Previous studies have shown that sleep disorders are closely related to metabolic disorders (17). Sleep deprivation has been linked to an increased risk of cardiovascular disease，such as hypertension, obesity, coronary artery disease, and stroke—these are all associated with morbidity and mortality (18). In addition, night shift work is correlated with a higher risk of MetS and high waist circumference versus daytime work (19). Men who work a permanent night shift show a higher risk of MetS than those with permanent day work, and this association could be mediated by visceral obesity (20). Therefore, this study explores whether night shift work or abnormal sleep duration in adulthood increases the risk of MetS in individuals born before or after the Tangshan earthquake.

## 2. Subjects and methods

### 2.1. Subjects

This research recruited miners from Tangshan, China who work for the Kailuan Mining Group. The workers change shifts at 8:00 am and midnight; hence, participants were classified into the day shift (from 8:00 am to 24:00) and the night shift (from 24:00 to 8:00 am the next day).

Participants were classified into three earthquake exposure groups. The infancy exposure group includes participants born between July 29, 1975 and April 28, 1976; they were aged three to 12 months when the Tangshan earthquake happened. The prenatal exposure group includes participants born between July 29, 1976 and April 28, 1977; these subjects experienced the earthquake prenatally. The control/no exposure group includes participants who were born between July 29, 1977 and April 28, 1978 or one to 1.9 years after the earthquake. All participants had lived in Tangshan since birth and agreed to participate in the study. Individuals whose mothers had infections, hypertension, epilepsy or seizures, diabetes, thyroid disease, or coronary heart disease during pregnancy were excluded. Individuals whose mothers experienced other traumatic events apart from the earthquake during pregnancy were excluded.

### 2.2. Subject evaluation

Participants received a standardized interview including demographics; smoking and drinking history; history of arterial hypertension, diabetes mellitus, and hyperlipidaemia; and the participants’ mothers’ conditions during pregnancy. Specially trained physicians made the interview and all examinations. Participants were classified into three groups according to smoking status: never smoked, ex-smoker, and current smoker. They were also divided into three groups according to drinking status: never drinks, ex-drinker, and current drinker. The Childhood Trauma Questionnaire (CTQ) and the Life Event Scale (LES) were adopted to assess traumatic events. Childhood trauma was defined as a CTQ score of 26 points or higher. An LES score of 32 or higher indicated a traumatic event in adulthood.

### 2.3. Physical measurements

The blood pressure of the right brachial artery was measured using a corrected mercury sphygmomanometer. Smoking, tea, and coffee were forbidden before this measurement, and workers sat quietly for 15 min before measure blood pressure. Three measurements were made consecutively at 1–2 min intervals, and the results were averaged. The evaluation of waist was as the body circumference midway between the lowest rib and the iliac crest as measured parallel to the floor with an inelastic tape 1measure without compressing the body tissue were made. The recording of measurements was made in centimetres with a sensitivity of 0.1 cm. The measurement of hip circumference was made similarly at the widest part of the hip.

### 2.4. Biochemical measurements

Blood was drawn between 7:00 am and 9:00 am on the same day as the physical examination. Blood was taken from the cubital vein of fasting subjects and placed in EDTA vacuum tubes. The 10-min centrifugal of samples was made at 3000 g at room temperature, and the collection of supernatants was performed. Fasting plasma glucose (FPG), triglycerides, low-density lipoprotein cholesterol (LDL-C), total cholesterol, and high-density lipoprotein cholesterol (HDL-C) were then measured. An oxidase approach was adopted to measure total cholesterol and triglycerides.

The definition of MetS was according to the guidelines for preventing and controlling type 2 diabetes in China (2017 Edition) (21). People with three or more of the following were diagnosed with MetS: (1) Abdominal obesity:the presence of central obesity (waist circumference ≥ 90 cm for men or ≥ 85 cm for women). (2) Hyperglycemia: fasting blood glucose ≥6.1 mmol/L or blood glucose ≥7.8 mmol/L 2 h after glucose load and/or diagnosed treated with diabetes. (3) Hypertension: SBP ≥130 mmHg or DBP ≥ 85 mmHg or treated with medication for hypertension. (4) Fasting triglyceride (TG) ≥ 1.70 mmol/L. (5) Fasting high-density lipoprotein cholesterol (HDL-C) < 1.04 mmol/L.

### 2.5. Sleep evaluation

The quality and duration of participants’ sleep were measured with the Pittsburgh Sleep Quality Index (PSQI). The PSQI consists of 7 subscales: the score ranges from 0 to 3 for each. Thus, the total points can vary from 0 to 21; a higher point represents poorer sleep quality. In this study, a PQSI score over seven is considered to indicate a sleep disorder.

### 2.6. Statistical analyses

SPSS 23.0 was adopted for all statistical analyses. The expression of measurement data was made using the mean and standard deviation (SD). Continuous variables for the three groups were compared with One-Way-ANOVA. Categorical variables for the three groups were compared with Chi-squared tests. Multivariate logistic regressions were used to identify factors that influence MetS. A two-sided value of p greater than 0.05 was of statistical significance.

## 3. Results

### 3.1. Demographic and baseline characteristics of three groups

**Table 1:** General information on workers of the three groups.

	No	Infant	Prenatal	Test value	*P*
N	Exposure 353	Exposure 244	Exposure 273		
Gender, *n* (%)					
Male	325 (92.1)	225 (92.2)	255 (93.4)	0.448	0.799
Female	28 (7.9)	19 (7.8)	18 (6.6)		
Age	37.5 ± 1.1	39.5 ± 0.5	38.5 ± 0.9	372.696	**<0.001**
Educational level, *n* (%)					
≤6 years	3 (0.8)	1 (0.4)	6 (2.2)	9.001	0.061
6 ~ 12 years	231 (65.4)	180 (73.8)	191 (70.0)		
>12 years	119 (33.7)	63 (25.8)	76 (27.8)		
Marital status					
Married	326 (92.4)	225 (92.2)	252 (92.3)	0.004	0.998
Others	27(7.6)	19(7.8)	21 (7.7)		
Family income per month for every person (RMB)					
≤2000	77 (21.8)	55 (22.5)	70 (25.6)	6.328	0.176
2000 ~ 5,000	244 (69.1)	177 (72.5)	177 (64.8)		
>5,000	32 (9.1)	12 (4.9)	26 (9.5)		
CTQ, *n* (%)					
No (<26)	34 (9.6)	20 (8.2)	25 (9.2)	0.363	0.834
Yes (≥26)	319 (90.4)	224 (91.8)	248 (90.8)		
LES, *n* (%)					
No (<32)	257 (72.8)	185 (75.8)	191 (70.0)	2.230	0.328
Yes (≥32)	96 (27.2)	59 (24.2)	82 (30.0)		
Smoke, *n* (%)					
Never-smoke	125 (35.4)	96 (39.3)	104 (38.1)	1.249	0.870
Ex-smoke	37 (10.5)	22 (9.0)	25 (9.2)		
Current-smoke	191 (54.1)	126 (51.6)	144 (52.7)		
Alchol use, *n* (%)					
Never-drinker	76 (21.5)	60 (24.6)	75 (27.5)	5.404	0.248
Ex-drinker	13 (3.7)	8 (3.3)	4 (1.5)		
Current-drinker	264 (74.8)	176 (72.1)	194 (71.1)		
Working shift, *n* (%)					
Day shift	242 (68.6)	158 (64.8)	196 (71.8)	2.961	0.228
Night shift	111 (31.4)	86 (35.2)	77 (28.2)		
Sleep duration, *n* (%)					
7 h	128 (36.3)	97 (39.8)	90 (33.0)	2.807	0.591
<7 h	123 (34.8)	84 (34.4)	103 (37.7)		
>7 h	102 (28.9)	63 (25.8)	80 (29.3)		
Sleep disorder, *n* (%)					
No	317 (89.8)	210 (86.1)	244 (89.4)	2.223	0.329
Yes	36 (10.2)	34 (13.9)	29 (10.6)		
MetS					
No	290 (82.2)	192 (78.7)	201 (73.6)	6.639	**0.036**
Yes	63 (17.8)	52 (21.3)	72 (26.4)		

Others marital status: Not married, divorce, remarriage and widowhood; CTQ: Childhood trauma questionnaire; LES: Life event scale. Bold values means *P* < 0.05.

Employees of the Kailuan Mining Group were scanned; 928 individuals satisfied the inclusion standards and agreed to take part in the research, and 58 additional individuals were excluded after the participants completed the questionnaire and the PSQI. Ten individuals were excluded because of missing data on sleep and earthquake exposure; 17 were excluded due to prenatal exposure to other stress; and 31 withdrew from the study because they chose not to undergo blood sample collection. There were 870 participants included in the analyses.

Of the 870 participants, 273 workers who experienced the earthquake as fetuses, 244 workers who experienced the earthquake as infants and 353 workers who did not experience the earthquake (Table 1). 187 (21.5%) met the diagnostic criteria for MetS. MetS was greatly more common in infancy exposure group (21.3%) and prenatal exposure group (26.4%; Table 1) than no exposure group (17.8%，*χ^2^* = 6.639, *p* = 0.036). There was a statistical difference in age among the three groups (*F* = 372.696，*P*<0.001; Table 1).

### 3.2. Mets incidence in earthquake exposure groups and in day vs. night shift workers in all three exposure groups

**Table 2:** Comparison the incidence of MetS between day shift and night shift in each group.

		No MetS	MetS	*χ^2^*	*P*
No exposure					
	Day shift	203 (83.9)	39 (16.1)	1.573	0.210
	Night shift	87 (78.4)	24 (21.6)		
Infant exposure					
	Day shift	133 (84.2)	25 (15.8)	**8.053**	**0.005**
	Night shift	59 (68.6)	27 (31.4)		
Fetus exposure					
	Day shift	145 (74.0)	51 (26.0)	0.045	0.833
	Night shift	56 (72.7)	21 (27.3)		

Bold values means *P* < 0.05.

All subjects were grouped by earthquake exposure, and the groups were compared. Among workers in the infant-exposure group, night shift workers had a higher risk of MetS than day shift workers (31.4% vs. 15.8%, *p* = 0.005; Table 2). In the unexposed group (21.6% vs. 16.1%, *p* = 0.210) and the prenatal exposure group (27.3% vs. 26.0%, *p* = 0.833 Table 2), night shift workers had slightly higher rates of MetS than day shift workers.

### 3.3. Multivariate logistic regression analysis of MetS risk factors

**Table 3:** Multivariate logistic regression analysis of risk factors of MetS.

	*B*	*P*	*OR*	*95%CI*
Gender				
Male				
Female	−0.999	**0.042**	**0.368**	**(0.140, 0.965)**
Smoke				
Non-smoker				
Ex-smoker	−0.082	0.806	0.921	(0.478, 1.776)
Current-smoker	0.419	**0.030**	**1.520**	**(1.042, 2.218)**
Earthquake exposure				
No				
Infant	0.245	0.247	1.278	(0.843, 1.937)
Fetus	0.497	**0.012**	**1.644**	**(1.115, 2.423)**
Sleep duration				
7 h				
<7 h	0.494	**0.015**	**1.638**	**(1.101, 2.437)**
>7 h	0.421	0.053	1.523	(0.994, 2.334)

Bold values means *P* < 0.05 or the difference is statistically significant.

Participant groups were compared using a multivariate logistic regression analysis with or without MetS as the dependent variable. Factors that including gender, smoking and alcohol status, sleep duration, sleep disorder，working shift and earthquake exposure were used as independent variables.

The multivariate analysis showed that male participants had a higher risk develop MetS than female participants (*p* = 0.042, *OR* = 0.368, *95% CI* = 0.140, 0.965; Table 3). Current smokers showed a higher risk (*p* = 0.030, *OR* = 1.520, *95%CI* = 1.042, 2.218; Table 2) of MetS. Participants who sleep fewer than 7 hours (*p* = 0.015, *OR* = 1.638, *95%CI* = 1.101, 2.437; Table 3) also showed a higher risk of MetS. Prenatal earthquake exposure was also correlated with an increased risk of MetS (*p* = 0.012, *OR* = 1.644, *95%CI* = 1.115, 2.423; Table 3).

## 4. Discussion

On July 28, 1976, a 7.8-magnitude earthquake struck Tangshan, China, instantly destroyed the entire city. The loss of traffic infrastructure, telecommunications, power, and water supply led to 242,000 deaths as well as serious injury to 164,000 residents. Fetuses and infants exposed to earthquakes are affected not only by the direct stress of the earthquake, but also by water and power outages, food shortages, and changes in living conditions after the earthquake. We found here that miners who were exposed to earthquake stress prenatally had a significantly higher risk of MetS. This may be related to the lack and imbalance of maternal nutrition after the earthquake. Animal research has shed light on the fundamental underlying mechanisms that the imbalance of specific amino acids and other nutrients could be the decisive factor in programming of MetS-related phenotypes (22). Additionally, deficiencies of micronutrients in pregnant mother rats, such as calcium, zinc and vitamin D, are linked to offspring MetS (22). Furthermore， prenatal stress may lead to abnormal methylation levels of genes associated with important pathways of embryogenesis in the placenta, possibly affecting fetal development, as well as the peripheral tissues of the mother and baby (23). Both animal and human studies have confirmed that epigenetic mechanisms are important modulators of the susceptibility of offspring exposed to stressors during pregnancy to chronic diseases, especially metabolic diseases (24, 25). The result of this study conforms to the findings of the Dutch Famine Birth Cohort Study, which observed that kids of pregnant women exposed to famine presented with various characteristics of MetS including obesity, dyslipidaemia, hypertension, and insulin resistance (8). However, previous studies on this topic have only examined one or a few characteristics of MetS. Here, we focus on subjects who clearly meet the MetS diagnosis criteria.

More importantly, our findings suggest that workers exposed to earthquake stress during infancy have a greatly higher risk of MetS, and this risk increases further for those who work night shifts. Wang and colleagues observed that exposure to the Great Chinese famine during infancy was correlated with an increased risk of MetS in adults in their 50s (12). Importantly, our subjects are younger (38 to mid-40s) than those examined by Wang and colleagues. Bayon and colleagues found that men working on permanent night shifts show a higher risk of MetS than those with permanent day work (20). Another inconsistent found that night shift work, and sleep duration per night and day had no association with the higher odds of having MetS (26). It is important to note that previous studies have focused on only one factor (early life adversity or adult sleep conditions) that may influence the risk of developing MetS. Specifically, our findings confirm that individuals exposed in infancy are more susceptible to MetS - especially if they experience sleep rhythm disturbances in adulthood.

The mechanism linking early-life exposure to environmental stress and MetS risk is poorly understood. However，compelling evidence shows that infancy are windows of particular sensibility to environmental clues which influence lifelong trajectories of health and disease (27). Early-life stress may cause a blunted cortisol response to social stress in later life, moreover, that the long-term adverse effects of early life stress may reach peak levels in adults (28). Furthermore，night shift work disrupted diurnal changes in hypothalamic–pituitary–adrenal (HPA) axis activity associated with cortisol arousal response (29). Continuous maladaptation of the HPA axis leads to various stress disorders such as MetS, hypertension, and diabetes mellitus (30). Previous animal research has displayed a link between early life stress and chronic cardiorenal metabolic pathologies including alterations to organ weight, affective state, and metabolites and/or metabolic paths (31). These conditions are further associated with adverse mental health results and metabolic illnesses including insulin resistance, cardiorenal syndrome, diabetes, and obesity (31).

As a microbial ecosystem, the gut microbiota exerts a significant effect on human health. The composition and function of the human gut microbiota develop over the first years of life, and a strong microbial population is established by age two (32). The gut microbiota encounters several perturbators in individuals who experience trauma during this period, e.g., unhealthy diet or drug exposure (33). If the microbiota cannot oppose these attacks, then a permanent variation happens, resulting in a new balance of microorganisms. This new balance may not be healthy, thus creating a condition called dysbiosis. In addition, it is unlikely that the negative impact of poor nutrition in early life is eliminated by healthy lifestyle elements during adulthood (34). Further research is required to identify the exact mechanisms underlying this association.

Female is a protective factor for MetS in our sample, consistent with other study. As shown in recent results demonstrated that males (6.1%) have nearly twice the prevalence of young-onset MetS compared with females (3.5%) (35). Compared with their study, our study had a higher incidence of MetS. On one hand is because the differences in diagnostic criteria of MetS and on other hand is because their study subjects were relatively young (between 18 and 40 years old). In our sample，females were less likely to work night shift than males (7.7% vs. 33.4%, *χ^2^* = 18.446, *P*<0.001). Females were also less likely to smoke than males (1.5% vs. 57.8%, *χ^2^* = 102.126, *P*<0.001).

More importantly, our findings suggest that workers who slept fewer than 7 h were more likely to develop MetS than those who slept 7 h. Previous studies showed that inadequate sleep is a risk element for obesity and diabetes (36). During sleep, the body restores and replenishes energy. Adipokines may exert an effect on mediating the close connection between sleep disorders and systemic metabolic disorders (17). As an adipocyte-derived peptide and a regulator of food intake and energy expenditure, Leptin is related to short sleep duration in the pathophysiology of obesity and accordingly type 2 diabetes (37). The growth of ghrelin levels after sleep loss has been determined in follow-up research (38). Ghrelin is a hormone generated in the oxyntic glands of the stomach and plays an orexigenic role. Hanlon and colleagues studied subjects who slept for only 4.5 h for four nights and found peak midday levels of the most abundant circulating endocannabinoid (eCB), 2-arachidonoylglycerol (2-AG), were elevated in a 24-h profile versus subjects who slept 8.5 h (39). Subjects with restricted sleep also experienced growing hunger and appetite and a reduced capacity of inhibiting snack intake (39). Inadequate sleep may increase the risk of MetS by causing changes in the amount and rhythm of these appetite-regulating hormones.

Consistent with previous studies, we found that current smokers had a higher risk of MetS. It was more likely that smokers show high insulin resistance than their non-smoking counterparts, and smoking cessation may protect against insulin resistance (40). A large sample study on alcohol consumption and MetS in South Korea found that, compared to complete abstinence, drinking less than 7 g/d reduced the risk of MetS, and drinking more than 7.1 g/d grew the risk of MetS (41). We found no relationship between alcohol consumption and MetS probably because we did not divide current drinkers into light and heavy drinkers.

## 5. Limitations

Other factors that were not included here such as diet and exercise may confound our analysis. These factors were excluded due to the difficulty of performing quantitative analyses on these confounding factors. The sex ratio of men and women in the current sample is unbalanced. Most subjects (92.5%) in this study were male. It is importantly that we corrected for sex as a confounding factor in multifactorial logistic regression (Table 3).

## 6. Conclusions and future research

Our results show that the risk of MetS is significantly higher in night shift workers exposed to earthquake stress during infancy than day shift workers. We also show that the presence of MetS was significantly higher in males than in females. Earthquake exposure during pregnancy is an independent risk factor for MetS. Smoking and sleeping less than 7 h have a higher risk of MetS than the control group. Future research should use larger sample size to explore hormonal indicators related to early-life stress such as HPA axis-related hormones as well as and biological markers related to the MetS such as orexin, which has been shown to be related to both the MetS and stress in recent studies. Thus, it is important to further investigate the effect of orexin on early-life stress and MetS in adulthood.

## Data availability statement

The original contributions presented in the study are included in the article/supplementary material, further inquiries can be directed to the corresponding author.

## Ethics statement

The studies involving human participants were reviewed and approved by the Ethics Committee of the First Hospital of Hebei Medical University (No 2014005). The patients/participants provided their written informed consent to participate in this study.

## Author contributions

XW and NL designed the paper. NL, RW, PH, WL, XZ, and YG performed the experiment. LW, MS, and CA analyzed the data. NL wrote the manuscript. XW, RW, and SB reviewed the manuscript. All authors contributed to the article and approved the submitted version.

## Funding

This study was supported by the S&T Program of Hebei (SG2021189), Project of Clinical Medical Research Center for Psychiatric and Psychological Disorders of Hebei Province (199776245D), Provincial Science and Technology Program of Hebei Province (21377711D), Hebei Medical University Clinical Research Innovation Team (2022LCTD-A1) and Introduce Foreign Intellectual Projects of Finance Department in Hebei Province (YZ202204). Medical Research Projects from Health Commission of Hebei Province (20210418).

## Conflict of interest

The authors declare that the research was conducted in the absence of any commercial or financial relationships that could be construed as a potential conflict of interest.

## Publisher’s note

All claims expressed in this article are solely those of the authors and do not necessarily represent those of their affiliated organizations, or those of the publisher, the editors and the reviewers. Any product that may be evaluated in this article, or claim that may be made by its manufacturer, is not guaranteed or endorsed by the publisher.
